# Collaborative Robotic Systems for Pre-Analytical Processing of Biological Specimens in a Medical Laboratory

**DOI:** 10.3390/diagnostics16071093

**Published:** 2026-04-04

**Authors:** Andrey G. Komarov, Pavel O. Bochkov, Arkadiy S. Goldberg, Vasiliy G. Akimkin, Pavel P. Tregub

**Affiliations:** 1 Moscow Scientific and Practical Center for Laboratory Research, Moscow 125424, Russia; 2 Department of Resource Management in Healthcare, N.A. Semashko National Research Institute of Public Health, Moscow 119435, Russia; 3 Central Research Institute of Epidemiology, Moscow 111123, Russia; 4 Department of Pathophysiology, Sechenov First Moscow State Medical University (Sechenov University), Moscow 119991, Russia; 5 Scientific and Educational Resource Center “Innovative Technologies of Immunophenotyping, Digital Spatial Profiling and Ultrastructural Analysis”, RUDN University, Moscow 117198, Russia

**Keywords:** collaborative robots, clinical laboratory, IVD management, efficiency indicators, healthcare

## Abstract

**Background/Objectives**: The increasing volume of laboratory testing and the tightening of quality standards have rendered automation tasks in medical laboratories highly relevant. Conventional total laboratory automation (TLA) systems demonstrate high throughput; however, their economic and organizational efficiency is often constrained by their complex integration and substantial implementation costs. In this context, collaborative robots (cobots) are attracting increasing attention due to their ability to perform pre-analytical and logistical tasks in close association with laboratory personnel. The objective of the present study was the systematic integration of commercially available cobots into the pre-analytical workflow of a large centralized laboratory. **Methods**: The implemented system incorporated a set of specialized modules, including decapping, barcode orientation and verification, analyzer loading, aliquoting, and specimen sorting, with bidirectional integration into the Laboratory Information System (LIS). The architectural design, control algorithms, and primary effects on labor input and operational turnaround time were evaluated. **Results**: The results demonstrated that the implementation of cobots into laboratory processes led to an 87% reduction in labor input, a 3.4% improvement in liquid aliquoting accuracy, and an overall improvement in nominal throughput, while requiring minimal personnel training. However, human operators performed the aliquoting procedure significantly faster than cobots, with an average speed advantage of 42.5%. **Conclusions**: The use of collaborative robotic systems in centralized medical laboratories appears promising due to their operational efficiency and flexibility compared to conventional automation platforms and manual workflows. The effect of the use of cobots on the quality/accuracy of the tests needs to be evaluated, and perhaps a larger study of multiple laboratories needs to be conducted to confirm the results are generalizable.

## 1. Introduction

Laboratory diagnostics have undergone rapid development over recent decades, in parallel with imaging and functional modalities for patient assessment [1,2]. The market for laboratory equipment and consumables is among the most dynamic and expansive in the medical sector, reaching 258.7 billion USD in 2023 with a compound annual growth rate of 6.9% [3]. Analytical data indicate that approximately 80% of clinical decisions depend on laboratory test results [4,5,6]. Thus, laboratory diagnostics constitutes a critical component of contemporary medicine and exerts a substantial impact on healthcare system economics.

At present, clinical laboratories are experiencing a technological revolution driven by the active implementation of innovative solutions, including artificial intelligence, computer vision, advanced software systems, robotics, and big data analytics [7,8]. Another key dimension in the evolution of laboratory medicine in recent decades concerns the transformation of the workforce. Many medical laboratories worldwide continue to face a pronounced shortage of clinical informatics and cybernetics professionals competent in biological, medical, engineering, and information technology [9].

In the context of technological advancement and workforce transformation, particular attention should be paid to robotic systems that are being actively deployed for laboratory automation [10,11]. One of the most promising branches of robotics development is collaborative robots (cobots), which constitute a new generation of systems specifically designed for safe human–robot interaction within a shared workspace [12]. In contrast to conventional industrial robots, cobots are equipped with dedicated safety features that enable them to operate in close proximity to laboratory staff without physical safety barriers [13].

It is important to emphasize that the pre-analytical phase of the laboratory testing process is the most critical in terms of error risk, with error rates as high as 70% reported [1,14,15]. Thus, automation of the pre-analytical workflow is of paramount interest, and robotic pre-analytical processing systems have been shown to significantly improve data quality compared to manual specimen handling [16,17].

Despite their high integration potential, there are virtually no commercially available collaborative robotic solutions that are specifically adapted to the operational needs of medical laboratories. This results in a substantial gap between the technological capabilities of modern robotics and the practical requirements of clinical laboratory services [18,19,20].

The objective of the present study was the systematic integration of commercially available cobots into the pre-analytical workflow of a large centralized laboratory. The implemented system incorporated a set of specialized modules, including decapping, barcode orientation and verification, analyzer loading, aliquoting, and specimen sorting, with bidirectional integration into the Laboratory Information System (LIS). The architectural design, control algorithms, and primary effects on labor input and operational turnaround time were evaluated. The results of this study may serve as a foundation for implementing collaborative robots in other centralized medical laboratories to enhance the efficiency of pre-analytical procedures.

## 2. Materials and Methods

### 2.1. Medical Laboratory Parameters

In this study, collaborative robots were integrated into the pre-analytical processing of biological specimens at the largest public centralized clinical laboratory in Russia—the Moscow Scientific and Practical Center for Laboratory Research of the Moscow City Health Department—from March 2022 to the present. Across all branches of the centralized laboratory, more than 120 million laboratory tests are performed annually (over 250,000 per day). The test menu of the laboratory complex includes more than 2000 different assays performed in-house (including hematology, clinical chemistry, immunochemistry, chemical microscopic analyses, cytology, molecular biology, microbiology, histology, toxicology, and genetic testing). A total of 720 analytical instruments are installed in the laboratories, and more than 1600 staff members are employed. Biological specimens for testing are sent to the laboratory complexes from 300 healthcare facilities in Moscow and its neighboring regions. The core Laboratory Information System (LIS) used across all branches of the clinical laboratory is the Efir LIS (INFORMATION SYSTEMS IN HEALTHCARE Ltd., Moscow, Russia).

### 2.2. Technical Specifications of Collaborative Robots

Two commercial collaborative robot models were selected for integration into laboratory workflows: GBT-C5A (Agilebot Robotics Co., Ltd., Shanghai, China) and JAKA Pro5 (JAKA Robotics Co., Ltd., Shanghai, China). The key technical specifications of the cobots are summarized in Table 1.

**Figure 1:**
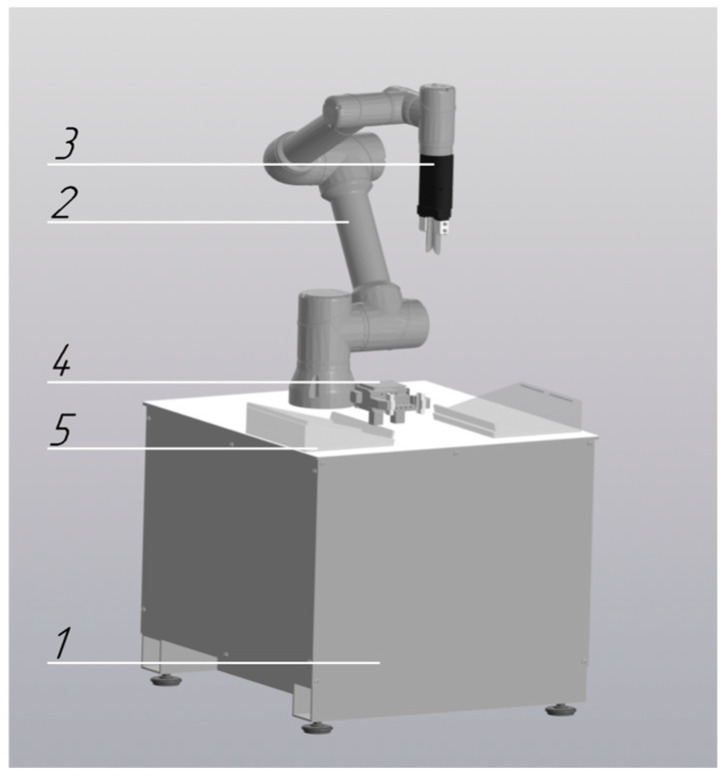
Robotic decapping module: 1—table platform; 2—collaborative robot; 3—movable gripper; 4—fixed gripper; 5—rack holder for sample tubes.

**Figure 2:**
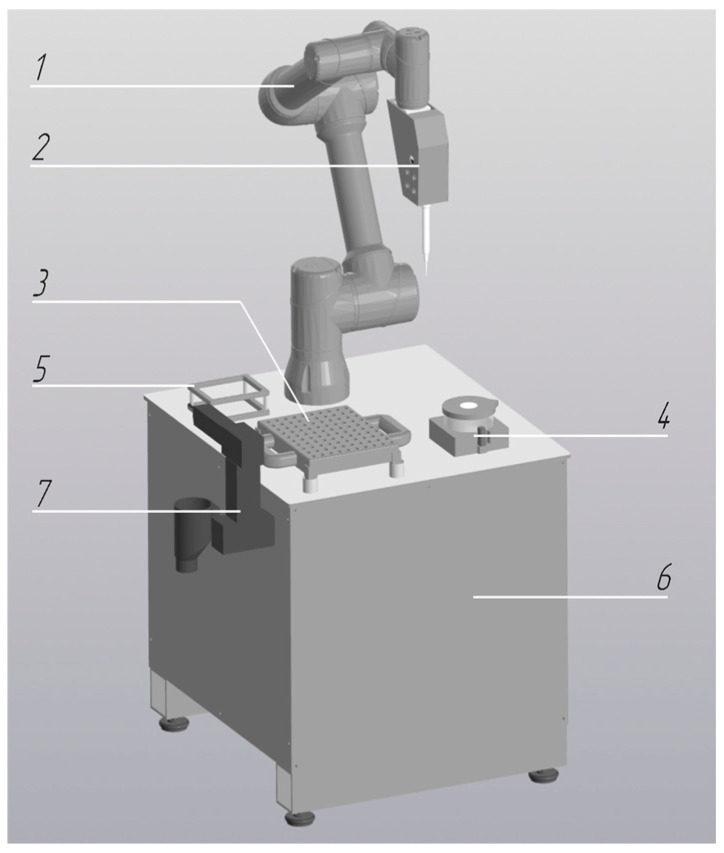
Robotic module for liquid aliquoting: 1—collaborative robot; 2—pipetting unit; 3—microtube platform; 4—reagent reservoir with level monitoring; 5—disposable tip storage box; 6—table platform; 7—waste container.

**Figure 3:**
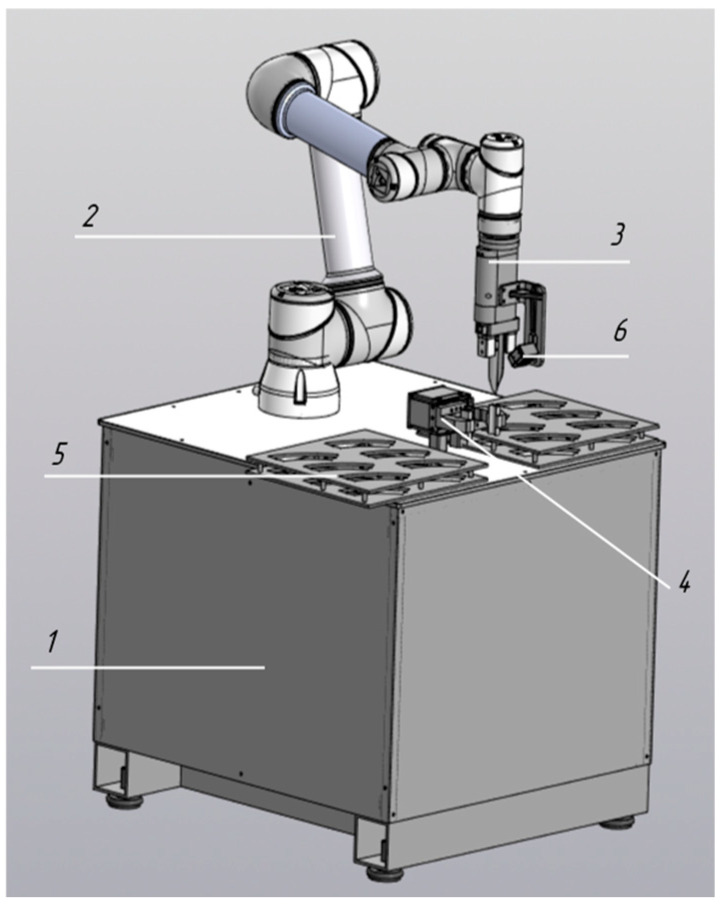
Robotic module for decapping and orientation: 1—table platform; 2—collaborative robot; 3—movable gripper; 4—fixed gripper; 5—rack holder for sample tubes; 6—industrial barcode scanner.

**Figure 4:**
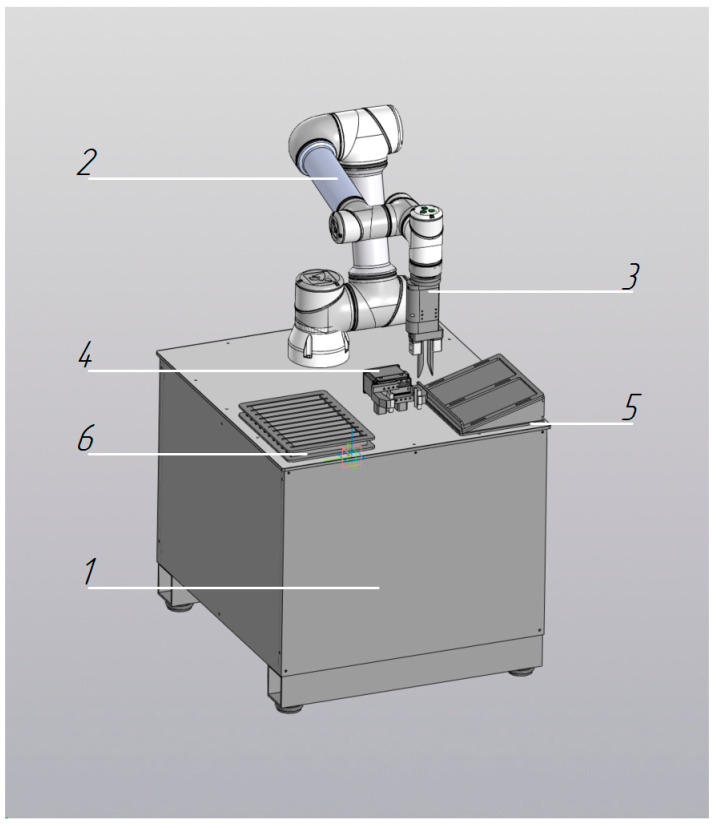
Robotic module for loading samples into the urine analyzer: 1—table platform; 2—collaborative robot; 3—movable gripper; 4—fixed gripper; 5—rack holder for sample tubes; 6—holder for analyzer-specific racks.

**Figure 5:**
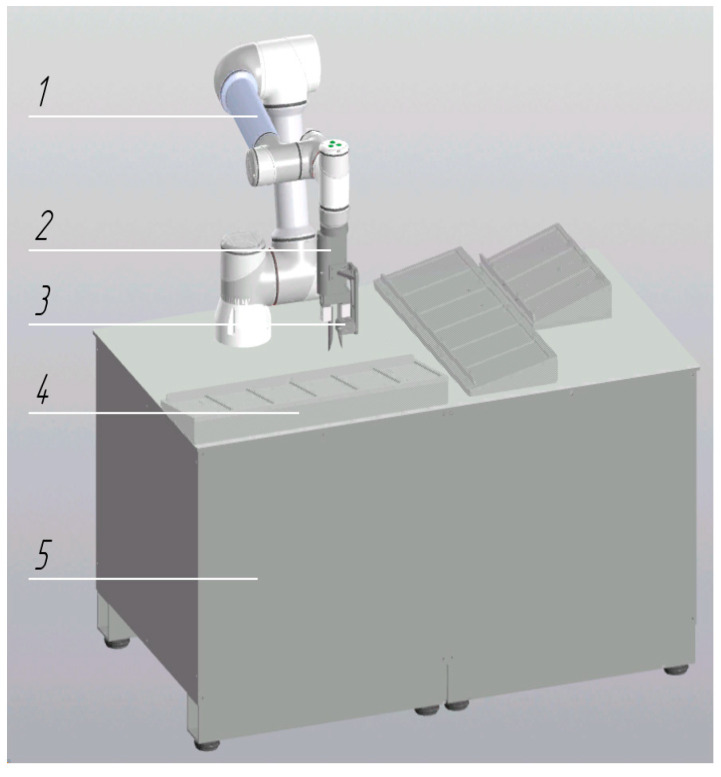
Robotic sorting module: 1—collaborative robot; 2—movable gripper; 3—scanner; 4—rack holders for sample tubes; 5—table platform.

**Figure 6:**
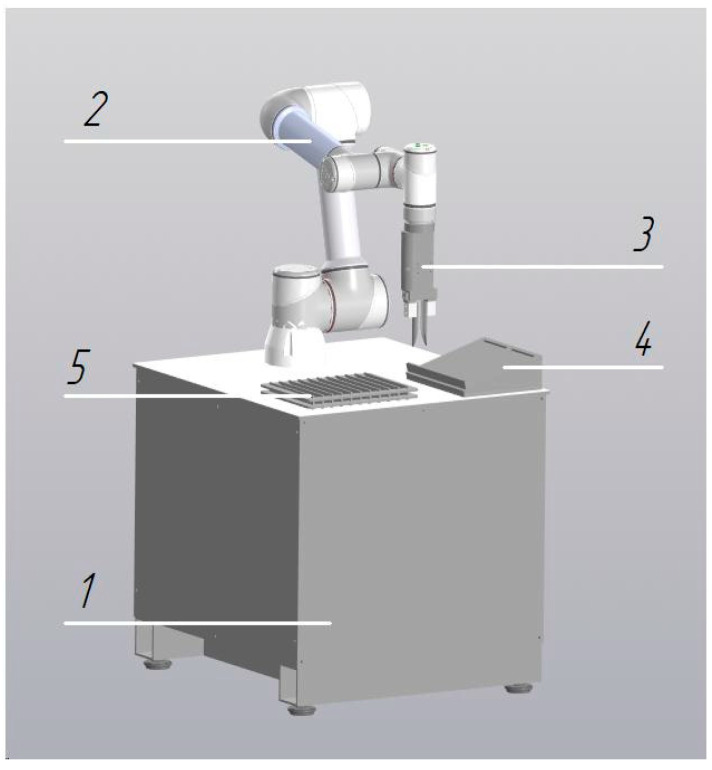
Robotic module for loading samples into a hematology analyzer: 1—support table (platform); 2—collaborative robot; 3—movable gripper; 4—rack holder for sample tubes; 5—rack holder for the analyzer’s reagent/sample racks.

During the integration of collaborative robots into the pre-analytical workflow of the centralized clinical laboratory, six cobot configurations were developed, each designed to perform distinct tasks and operations:

- Robotic Decapper (*n* = 31 units): Designed for automated tube cap removal without displacement of primary sample tubes from standard 50-position racks (Sarstedt, Nümbrecht, Germany) (Figure 1). Operational throughput is up to 300 specimens per hour.
- Robotic Aliquoter (*n* = 1 unit): Designed for precise dispensing of liquids into 2 mL microtubes (Figure 2). Operational throughput is up to 750 specimens per hour.

- Robotic Decapper–Orienter (*n* = 1 unit): Designed for automated tube cap removal without relocating sample tubes from analyzer rack holders, with correct barcode orientation (Figure 3). Operational throughput is up to 200 specimens per hour.
- Robotic Loader (Urine Analyzer (*n* = 13 units)): Designed for transferring urine sample tubes from standard 50-position racks (Sarstedt) to analyzer-specific rack holders, including tube mixing, decapping, and placement of loaded racks into the analyzer (Figure 4). Operational throughput is up to 200 specimens per hour.
- Robotic Sorter (*n* = 2 units): Designed for sorting sample tubes among standard 50-position racks (Sarstedt) based on Laboratory Information System (LIS) algorithms (Figure 5). Operational throughput is up to 450 specimens per hour.

- Robotic Loader (Hematology Analyzer (1 unit): an automated pre-analytical robotic system designed for the transfer of primary blood collection tubes from standard 50-position tube racks (Sarstedt) into analyzer-specific rack carriers of hematology analyzers, with subsequent automated placement of the loaded racks into the analytical module (Figure 6). Analytical throughput is up to 200 specimens per hour.

### 2.3. Description of Collaborative Robot Algorithms at the Pre-Analytical Stage

All software application algorithms were designed to enable robotic systems to efficiently perform the full range of standard operations required for pre-analytical procedures, with the capability to partially or fully replace manual labor and other commercial pre-analytical sample preparation systems (e.g., commercial sorters). Detailed descriptions of action sequences within repetitive cycles of pre-analytical procedures are provided in the Supplementary Materials (Table S1).

### 2.4. Comparison of Human- and Collaborative Robot-Controlled Procedures

To evaluate the effectiveness of the systemic integration of serial collaborative robots (cobots) at the pre-analytical stage in a large centralized laboratory, a time–motion study was conducted to compare traditional manual execution of pre-analytical procedures (decapping, loading into analyzers, sorting, aliquoting) to the implementation of cobots in the workflow. Time measurements were performed by two independent researchers over three days, with procedure execution times recorded by three laboratory staff members with varying levels of expertise.

To minimize the influence of subjective factors and increase the accuracy of the results, all participants received standardized instruction on procedure execution to ensure consistent conditions and a uniform approach. All laboratory equipment and robotic control applications were verified and calibrated according to unified standards and protocols prior to the experimental sessions. Additionally, before the comparative time–motion study, equipment calibration and functionality testing were conducted to exclude errors related to technical malfunctions. Data recording and management were performed using identical hardware and software tools, eliminating potential variability in information processing.

Based on the results of the time–motion studies analyzing traditional manual execution of pre-analytical procedures, standards were implemented for performing various operational tasks (including pre-analytical processing). These standards were expressed in Standardized Units of Labor (SUL). This parameter is advantageous for the objective quantification of labor inputs within the healthcare sector. Utilizing these data, estimation of medical procedure costs, budget allocation, and personnel planning were conducted. Representing labor time expenditure in SUL facilitates the standardization of workload through chronometric analysis of workflow procedures, expert assessment, and the application of various coefficients. In our study, these units were defined as 10 min blocks.

Six laboratory staff members performed the manual procedures for the comparative study. Participants were randomly selected from among laboratory personnel with professional experience ranging from 2 to 20 years. This approach ensured diversity in skill level, allowing for a more objective assessment of the impact of the new robotic technology and increasing the representativeness of the data. All participants had equivalent educational qualifications and documentation of professional training.

For the comparative assessment of accuracy and speed in liquid aliquoting, six series of measurements were conducted, each involving twenty microtubes (2 mL volume). For the calculation of the sample size necessary and sufficient to ensure adequate statistical power, an online calculator (https://www.sealedenvelope.com/ accessed on 13 February 2026) was utilized. The predetermined statistical power was 90%, with a type I error rate (alpha level) of 5% [21]. Aliquoting was performed using a single-channel Eppendorf pipette (Hamburg, Germany). The precision of microtube filling (using 0.9% NaCl solution) was evaluated via laboratory balances, and the total time required to complete each aliquoting series was recorded. The experimental series were randomized through alternation of operators and collaborative robots (cobots). Each series was conducted on separate days by six distinct cobot operators and six different laboratory personnel.

Standards for the number of operations, accounting for labor time spent on pre-analytical procedures (decapping, analyzer loading, sorting, aliquoting), were established by measuring no fewer than ten repetitions of the same operation and calculating the mean.

### 2.5. Collaborative Robot Software

The robot controller, supplied as part of the collaborative robot system, uses a motion control system that manages trajectory, acceleration/deceleration, positioning, and speed, maintaining a Tool Center Point (TCP) repeatability error of 0.02 mm. Communication between the robot controller and the PC server is established via the Transmission Control Protocol/Internet Protocol (TCP/IP).

The controller communicates with peripheral devices, such as the gripper, using the Modbus RTU protocol. It features a flexible system of string and numeric registers that can be dynamically modified and read during program execution through the Python SDK (v. 7.3.0) provided by the manufacturer, offering a high-quality, intuitive integrated development environment (IDE) for robot programming. The system includes a large number of available analog and digital inputs and outputs required for connecting peripherals and sensors.

This architecture enables the integration of the robot into a unified process control and dispatching system, allowing for the implementation of a universal process control solution in which the software code of the collaborative robot, microcontrollers, machine vision cameras, peripheral devices, sensors, and operator interface represents a set of instructions and checks continuously executed under varying initial conditions, depending on commands from the unifying program code running on a local or remote server.

### 2.6. Statistical Analysis

Statistical analysis was performed using Statistics for Windows 10.0 (StatSoft, Tulsa, OK, USA). To determine whether statistically significant differences existed in the procedure execution times or aliquoting accuracy between the two methods, the nonparametric Mann–Whitney U test was applied when the data were not normally distributed, and Student’s *t*-test was used when a normal distribution was observed across all samples. The normality of the distribution was assessed using the Shapiro–Wilk test. Averaged data in tables are presented as mean (M) ± standard deviation (SD).

## 3. Results

### Effectiveness of Collaborative Robot Implementation in Pre-Analytical Sample Processing

Pilot-industrial operation of collaborative robot technology was conducted over a 90-day period. During this time, more than 4.77 million samples were processed using cobots in one branch of the centralized medical laboratory, and a comparative analysis of labor productivity was conducted to evaluate the effectiveness of implementing the new technology. The sample volume reflects the typical throughput of biological material received during this period in the laboratory unit, ensuring that the sample size represents real laboratory workflow conditions analogous to the current study.

The results of the systematic integration of commercially available cobots into the pre-analytical workflow (decapping, barcode orientation and verification, analyzer loading, aliquoting, and specimen sorting) of the large centralized laboratory demonstrated that the mean difference between the labor productivity coefficients yielded for the traditional manual method and the automated pre-analytical system based on collaborative robots was 87% (Table 2). The frequency of procedures such as manually decapping and loading samples into analyzers was reduced by a factor of 30, while re-sorting occurred almost entirely without personnel involvement. The lowest potential for labor reduction was observed in the aliquoting procedure due to the need for microtubes and tips to be installed by operators.

The implementation of a collaborative robot (cobot) for liquid aliquoting procedures demonstrated a significant improvement in the precision of dispensing liquids into microtubes compared to manual human operation (Table 3), while concurrently revealing a substantial 42.5% reduction in procedural throughput (*p* < 0.001).

## 4. Discussion

The automation of procedures performed in medical laboratories has advanced significantly over recent decades. Initially based on simple mechanical devices, it has evolved into complex integrated systems [18,22]. Modern automation systems aim to integrate pre-analytical, analytical, and post-analytical processes into a single technological workflow [17,23], and leading laboratory equipment developers are actively working on solutions designed for full laboratory automation (TLA) [18,19].

The implementation of TLA systems demonstrates significant advantages, including a 30–50% reduction in turnaround time, a 60–80% decrease in errors, and improved workplace ergonomics [24,25]. An economic analysis of TLA implementation in a multidisciplinary hospital laboratory revealed a payback period of 4.75 years with substantial productivity gains [25]. Key economic benefits include a 40–60% reduction in labor in the pre-analytical phase, 50–70% fewer repeat analyses, optimized use of reagents and consumables, and a 30–50% reduction in sample turnaround time (TAT).

However, full laboratory automation is not always economically feasible and has significant drawbacks, such as challenging technical integration for certain analyzer models, risk of disruption to the diagnostic workflow in case of track system failures, dependency on a single vendor, and reduced process flexibility. Full automation can replace human operators in tasks such as sorting and sample preparation, pipetting, inter-analyzer transport, and measurement execution. In contrast, partial automation robotic systems perform only repetitive tasks while laboratory personnel maintain oversight of the process. In the case of partial automation, laboratories can implement localized automated or robotic solutions for specific processes, such as container reception, sorting, or sample logistics. This can be effectively achieved using collaborative robots, offering high-performance yet autonomous workstations for the automation of manual processes in clinical and research laboratories [26].

It should also be noted that debate continues among roboticists regarding the feasibility of collaborative robotics [27,28]. The main advantage of human–robot collaboration lies in increased productivity with relatively low effort required for integration. Personnel require only brief training to operate, pause, or reset cyclic robotic applications if errors should occur. This approach is applicable to medical laboratories, as collaborative robots represent an intermediate solution between the complexity, cost, and performance of TLA systems and the flexibility, adaptability, and low cost of manual processes [29,30].

The COVID-19 pandemic has accelerated the adoption of robotic solutions in healthcare [7,31]. During this period, collaborative robotics technologies were rapidly implemented for disinfection, diagnostics, transport, and sample preparation. In this regard, several studies have demonstrated the successful application of robots in various aspects of medical laboratory operations:

- Logistics and delivery of medications, consumables, and equipment within multi-story hospital complexes [32,33];
- Serological testing for SARS-CoV-2 antibodies with partially automated procedures using a dual-arm collaborative anthropomorphic robot [7];
- Precision microbiological tasks, including colony isolation with sub-millimeter accuracy [12];
- Microplate sample processing with a positioning accuracy of ±1.2 mm [34].

A recent example of collaborative robot implementation in laboratory diagnostics is the dual-arm system, deployed in 2022 for SARS-CoV-2 antibody testing [7]. Its implementation increased laboratory throughput by 66% and reduced manual pipetting operations by 62%.

In 2022, Khalapyan et al. proposed an integrated system with two robots: a parallel delta robot for dispensing and a collaborative serial robot for tube handling. Equipped with a camera and computer vision algorithms, the system automatically identifies liquid boundaries in tubes and performs blood and plasma aliquoting [35].

Practical experience shows that successful automation requires comprehensive personnel training, a culture shift toward innovation, clear role definition between humans and robots, gradual pilot implementation, and stepwise scaling. These principles are equally applicable to collaborative robotics.

However, the literature lacks documented evidence of successful large-scale implementations of collaborative robots (cobots) during the pre-analytical phase in large centralized laboratories. Moreover, the efficacy of cobot integration for reducing the workload of laboratory personnel remains unknown. Our study aimed to address this gap and may serve as a foundation for implementing collaborative robotic systems in other centralized medical laboratories to enhance the efficiency of pre-analytical laboratory procedures.

Our results demonstrate that systematic integration of commercially available cobots into the pre-analytical workflow (decapping, barcode orientation and verification, analyzer loading, aliquoting, and specimen sorting) of a large centralized laboratory reduces labor efforts in pre-analytical sample processing by 87%, improves liquid aliquoting accuracy by 3.4%, and results in an overall improvement in nominal throughput while requiring minimal personnel training. However, human operators performed the aliquoting procedure significantly faster than cobots, with an average speed advantage of 42.5%. Additionally, cobots reduce risks of misidentification, barcode damage, and sample contamination and enhance biosafety [36]. Deployment is currently being scaled across six laboratory branches.

The use of a cobot for liquid aliquoting procedures demonstrated higher accuracy in dispensing liquids into microtubes compared to human operators. This primarily indicates the superior precision of the cobot’s dispensing system relative to manual handling. However, human operators achieved faster execution speeds in performing liquid aliquotting procedures. These results suggest that human operators exhibit inherent inaccuracies in dispensing precision when striving for higher operational speed metrics.

Compared to pre-analytical automation systems that are commercially available in Russia (e.g., Sarstedt, Roche, Beckman Coulter), collaborative robots are substantially more cost-effective: the cost is ≈$43,000 per robot with required tooling and installation versus ≈$209,000–$376,000 for one AU2550 automated sample sorter (Beckman Coulter, Brea, CA, USA), representing a cost ratio of 4.9–8.7:1. The maximum nominal throughput is 450 samples/hour for the cobot versus 1200 samples/hour for commercial sorters (ratio 2.7:1).

It is also important to note that the total cost of ownership (TCO) for a single collaborative robot (cobot) over its entire service life (30,000 operating hours, equivalent to approximately 7 years) in our institution amounts to $24,877. This costs encompasses expenses for technical maintenance ($2968), consumables and spare parts ($115), integration and validation ($371), and tooling production costs ($327) as well as internal software-development costs ($371). Comparable expenditures for the AU2550 clinical chemistry analyzer over a 7-year period total $124,984, which represents a 4.9 increase. In addition, cobots offer superior flexibility in terms of their rapid reconfiguration and programming for different tasks. These results indicate both practical efficiency and economic potential for collaborative robots compared to traditional automation and manual methods.

The limitations of this study include the small number of repetitions performed for assessing aliquoting speed and precision and the absence of comparative data on the effect of cobots on pre-analytical issues (e.g., sample misidentification, barcode damage, hemolysis, clots, insufficient volume, contamination), as measured by scan failure rates and manual handling frequency. Caution should be exercised when extrapolating these results to laboratories with differing workflow structures, operation volumes, or pre-analytical algorithms.

The integration of diverse systems remains a major challenge in collaborative robot implementation [37,38]. Key technical issues include incompatibility between software from different manufacturers, the lack of standardization of robot and laboratory interfaces, unreliable communication protocols in critical applications, and difficulties in scaling solutions for laboratories of varying sizes.

Resistance to change among personnel is another important challenge [39]. These concerns relate to potential skill loss, workforce reduction, and distrust of advanced engineering, digital, and intelligent technologies. In our study, these challenges were addressed via phased implementation of the cobots, parallel staff training, and redistribution of labor to more specialized tasks, minimizing resistance to technological modernization. Cobots are now an integral part of routine laboratory workflow.

Many laboratories worldwide face a shortage of cybernetics specialists with combined biological, medical, engineering, and IT expertise [9]. Such personnel are crucial for digital development, the integration of intelligent systems, and the automation of laboratory diagnostics.

Patient safety and quality of care remain paramount in any robotic implementation. Standards for human–machine interaction (UNI EN ISO 10218-2:2025), validation of automated laboratory processes, protection of patient data in AI systems, and liability for errors in automated diagnostics must be carefully considered.

Future generations of collaborative robots are likely to feature close integration with AI systems [40,41]. Social robotics may enhance interaction between medical personnel and robotic systems [13,42]. Promising directions for future developments include natural voice and gesture interfaces, adaptive learning of operator preferences, emotional intelligence in robots to improve staff comfort, collaborative decision-making in complex diagnostics, and improved clinical decision support algorithms.

**Figure 7:**
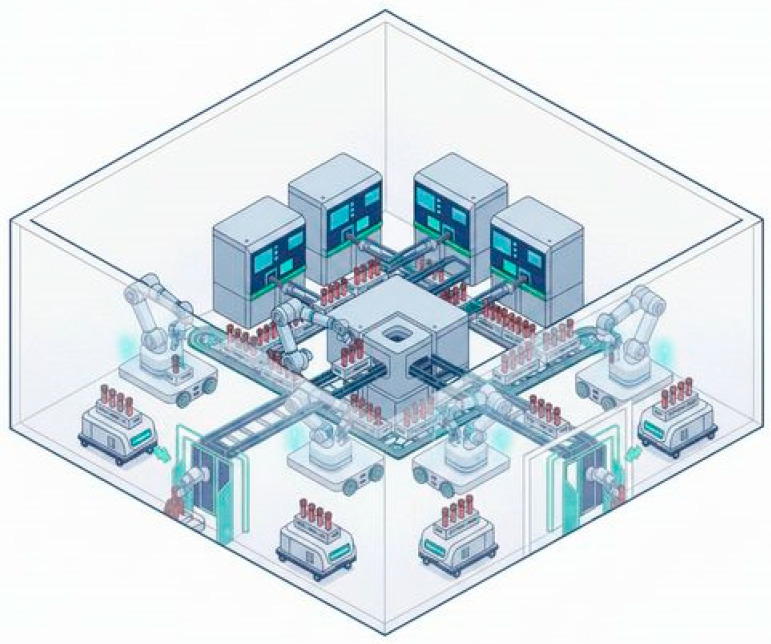
Schematic representation of an autonomous laboratory diagnostic complex.

The broader implementation of collaborative robotics in medical laboratories may involve the enhanced integration of engineering, digital, and intelligent solutions. For example, mobile robotic carts for sample transport in large hospitals and laboratories rely on computer vision and intelligent navigation, while cobots for pre-analytical processing and sorting with video-analytic modules have high economic potential. Such solutions move healthcare closer to the development of fully autonomous laboratory diagnostic complexes (Figure 7).

## 5. Conclusions

The implementation of collaborative robots in standardized procedures holds significant potential for transforming laboratory medicine, particularly in the automation of pre-analytical processes. Our practical experience with cobots in a centralized laboratory demonstrates that the technology has reached a level of maturity sufficient for widespread practical application.

The key advantages of implementing collaborative robots include improved quality and reproducibility of laboratory processes, reduced labor costs, and decreased risk of errors and biosafety breaches. Analyzing the systematic integration of commercially available cobots into the pre-analytical workflow (decapping, barcode orientation and verification, analyzer loading, aliquoting, and specimen sorting) of a large centralized laboratory confirms a tangible reduction in labor efforts within centralized clinical diagnostic laboratories.

Areas for future research include deep integration with artificial intelligence, the development of self-learning systems, and the creation of fully autonomous laboratory platforms. The successful realization of this potential will require interdisciplinary collaboration among specialists in medicine, robotics, AI, and healthcare management. The effect of the use of cobots on the quality/accuracy of the tests needs to be evaluated, and perhaps a larger study of multiple laboratories needs to be conducted to confirm the results are generalizable.

At the same time, it can be argued that practical implementation of collaborative robotic systems should proceed gradually, with careful consideration of the specific characteristics and profile of each laboratory, staff readiness, and the organization’s economic capacity. Under these conditions, collaborative robots have the potential to become a key factor in enhancing the efficiency and quality of laboratory diagnostics in the near future.

## Figures and Tables

**Table 1:** Technical specifications of collaborative robot models.

Parameter	GBT-C5A (Agilebot)	JAKA Pro5 (JAKA)
Service Life (operating hours)	30,000	30,000
Dimensions of the Working Area	2 m^2^	2 m^2^
Degrees of Freedom	6	6
Working Radius	933 mm	954 mm
Effective Payload	5 kg	5 kg
Ingress Protection (IP) Rating	IP67	IP54
Cleanroom Classification (ISO 14644-1:2015)	Class 4	Class 5
Teaching Method	Personal Computer (PC), Mobile Terminals (Tablet/Smartphone)	Personal Computer (PC), Mobile Terminals (Tablet/Smartphone)
Angular Range of Motion	J1: 720° (−360° to +360°), J2: 720° (−360° to +360°), J3: 720° (−360° to +360°), J4: 720° (−360° to +360°), J5: 720° (−360° to +360°), J6: 720° (−360° to +360°)	J1: 540° (−270° to +270°), J2: 280° (−50° to +230°), J3: 230° (−115° to +115°), J4: 350° (−85° to +265°), J5: 540° (−270° to +270°), J6: 540° (−270° to +270°)
Maximum TCP Speed	3 m/s	3 m/s
Positional Repeatability	0.02 mm	0.02 mm
Real-Time Program Editing	Yes	No
SDK-Based Control	Present	Present
End-Effector (Gripper) Control	RS-485	RS-485
Compliance with Industry Safety Standards (including biosafety)	Conforms to ISO 15190:2020	Conforms to ISO 15190:2020

**Table 2:** Comparative assessment of laboratory staff labor productivity coefficients before and after implementation of collaborative robots in pre-analytical sample processing.

			Labor Productivity Coefficients, SULs		
Pre-Analytical Procedure	Standard, Manual Operations/h	Average Number of Operations per Day, M ± SD	Before Cobot Implementation, SUL	After Cobot Implementation, SUL	Δ, %
Sample Check-In	600	53,285 ± 7779	532.85 ± 77.8	266.43 ± 78.6 *	50%
Tube Decapping	600	41,893 ± 6116	418.93 ± 61.2	13.96 ± 4.1 *	97%
Loading Samples into Urine Analyzers	600	5112 ± 746	51.12 ± 7.5	10.22 ± 3.0 *	80%
Loading Samples into Analyzers (Biochemical, Hematological, Immunochemical)	600	36,022 ± 5259	360.22 ± 52.6	12.01 ± 1.2 *	97%
Preparation of PCR Transport Media (Solution Aliquoting)	1200	500 ± 73	2.5 ± 0.73	2.0 ± 0.6 **	20%
Re-Sorting of Samples (Post-Analysis)	120	20,707 ± 3023	1035.35 ± 30.2	6.9 ± 2.0 *	99%
				Average (M ± SD)	80 ± 29%

Note: The labor productivity coefficient is presented in standard units of labor (SUL) and calculated using the following formula: (number of operations per day/standard) × 6. One SUL corresponds to 1/6 of an hour, or 10 min. *, statistically significant difference compared with labor productivity before the implementation of cobots at *p* < 0.001; **, statistically significant difference compared with labor productivity before the implementation of cobots at *p* < 0.05. M, mean; SD, standard deviation.

**Table 3:** Comparative assessment of precision and speed of liquid aliquoting by a collaborative robot and human operator.

№ Measurement Series	Operator	Number of Samples, *n*	Aliquoted Liquid Mass, g	M ± SD	Completion Time per Series, s	M ± SD
#1	Cobot #1	20	0.705	0.705 ± 0.003 *	130	133 ± 5.16 *
#2	Cobot #2	20	0.704		130	
#3	Cobot #3	20	0.705		140	
#4	Cobot #4	20	0.705		130	
#5	Cobot #5	20	0.704		130	
#6	Cobot #6	20	0.705		140	
#1	Human #1	20	0.728	0.683 ± 0.029	80	77 ± 5.16
#2	Human #2	20	0.662		80	
#3	Human #3	20	0.652		70	
#4	Human #4	20	0.668		80	
#5	Human #5	20	0.703		80	
#6	Human #6	20	0.688		70	

Note: *, difference compared with human results at *p* < 0.001. M, mean; SD, standard deviation.

## Data Availability

The original contributions presented in this study are included in the article/Supplementary Materials. Further inquiries can be directed to the corresponding author.

## Supplementary Materials

The following supporting information can be downloaded at: https://www.mdpi.com/article/10.3390/diagnostics16071093/s1, Table S1: Collaborative Robot Algorithms; Video S1: the work of the cobot-decapper; Video S2: the work of the cobot-orientator; Video S3: the work of the cobot-aliquoter; Video S4: the work of the cobot-sorter.

## Abbreviations

The following abbreviations are used in this manuscript:

SUL	the standard unit of labor
TAT	Turnaround time
TCP	Tool Center Point
SDK	Software Development Kit
TLA	total laboratory automation
IDE	integrated development environment
AI	Artificial intelligence
