# Supplementary material for: Collaborative Robotic Systems for Pre-Analytical Processing of Biological Specimens in a Medical Laboratory
Source: Diagnostics (Basel). 2026 Apr 4;16(7):1093. doi: 10.3390/diagnostics16071093 (PMC13073645; doi:10.3390/diagnostics16071093)
Supplement: Supplementary file 1 [file diagnostics-16-01093-s001.zip › Table S1.pdf]

Kobot's appearance	Collaborative Robot Models	Description of Collaborative Robot Algorithms at the Pre-analytical Stage
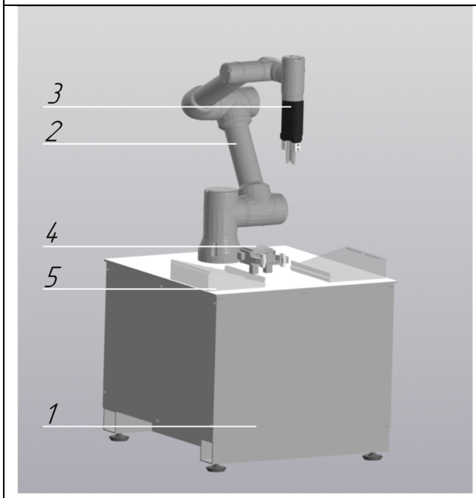	Robotic Decapper	1. Initialization of the collaborative robot and grippers, followed by positioning of the robot at the start location;<br>2. Movement of the robot toward a closed sample tube;<br>3. Closure of the movable gripper pins under robot command;<br>4. Verification of tube presence within the gripper pins – if a tube is absent, the gripper opens, and the robot proceeds to the next tube. In the case of two consecutive missing tubes, the robot moves to the next rack or proceeds to cycle termination;<br>5. Movement of the robot toward the fixed gripper;<br>6. Commanding closure of the fixed gripper by the robot;<br>7. Movement of the robot along the tube axis while simultaneously commanding rotation of the movable gripper, thereby opening the tube;<br>8. Discharge of the tube cap into the waste container;<br>9. Return of the opened tube to its original position.
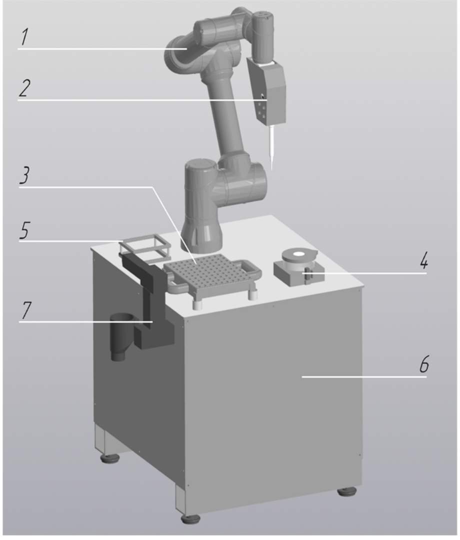	Robotic Aliquoter	1. Robot initialization: the collaborative robot is initialized and moved to its starting position.<br>2. Liquid level verification: a dedicated sensor monitors the liquid level in the container. If the liquid is insufficient, the program halts execution and the robot signals the operator.<br>3. Tip attachment: a disposable tip is mounted on the mechanical dispenser, which is secured to the moving unit of the collaborative robot.<br>4. Movement to the source container: the robot moves to the container with the liquid for aliquoting; the tip is lowered to a predefined depth.<br>5. Liquid aspiration: the robot sends a command to the programmable logic controller (PLC) to activate the stepper motor shaft, which rotates the eccentric cam and aspirates the liquid into the mechanical pipette from the source container.<br>6. Movement to the target microtube: the robot moves to the next well of the microtube pallet according to the cycle iteration number.<br>7. Liquid dispensing: the robot sends a command to the PLC to reverse the rotation of the stepper motor shaft, rotating the eccentric cam to dispense the liquid from the mechanical pipette into the microtube.<br>8. Cycle repetition or tip disposal: if a multiple-use tip protocol is set, the cycle repeats. For single-use tips, the tip is discarded into a disposal tray, and the process returns to step 3
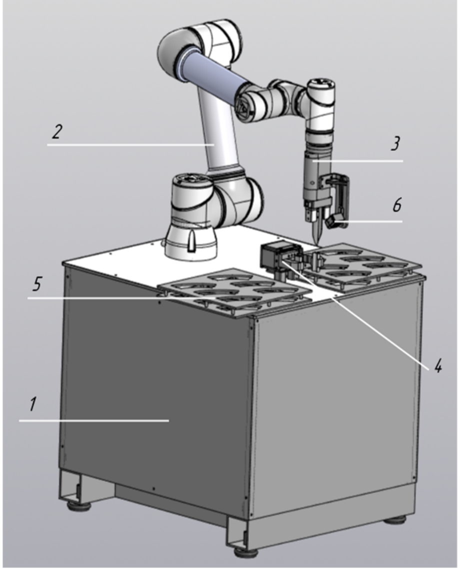	Robotic Decapper-Orienter	1. Robot initialization: the collaborative robot is initialized and moved to its starting position.<br>2. Movement to the closed tube: the robot approaches a closed microtube.<br>3. Grip closure: the movable gripper pins close around the tube upon robot command.<br>4. Tube presence verification: the system checks for the presence of the tube in the gripper. If the tube is absent, the gripper opens, and the robot moves to the next tube. In the absence of two consecutive tubes, the robot proceeds to the next rack or to the end of the cycle.<br>5. Movement to stationary gripper: the robot transfers the tube to the fixed gripper.<br>6. Tube rotation and barcode scanning: the robot rotates the tube and commands the scanner to read the barcode.<br>7. Barcode processing and orientation: the scanner reads the tube barcode, determining its orientation, and sends a command to rotate the tube so that the barcode is aligned to the predefined position.<br>8. Stationary gripper closure: the robot commands the stationary gripper to close.<br>9. Tube opening: the robot moves the tube along its axis while sending a rotation command to the movable gripper, thereby opening the tube.<br>10. Cap disposal: the tube cap is discarded into the waste tray.<br>11. Return of the open tube: the robot returns the opened tube to its original position while maintaining the oriented position

<table>
<tr>
<td>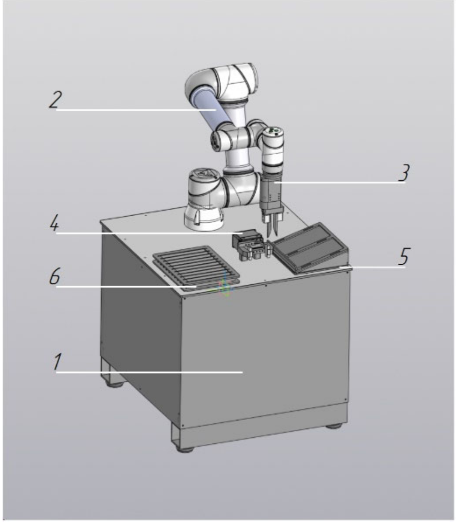
</td>
<td>Robotic Loader– URIT</td>
<td>1. Robot initialization: the collaborative robot is initialized and moved to its starting position.
2. Movement to the closed tube: the robot approaches a closed microtube.
3. Grip closure: the movable gripper pins close around the tube upon robot command.
4. Tube presence verification: the system checks for the presence of the tube in the gripper. If the tube is absent, the gripper opens, and the robot moves to the next tube. In the absence of two consecutive tubes, the robot proceeds to the next rack or to the end of the cycle.
5. Sample mixing: the robot mixes the contents of the tube by rotating and inverting it.
6. Movement to stationary gripper: the robot transfers the tube to the fixed gripper.
7. Stationary gripper closure: the robot commands the stationary gripper to close.
8. Tube opening: the robot moves the tube along its axis while sending a rotation command to the movable gripper, thereby opening the tube.
9. Cap disposal: the tube cap is discarded into the waste tray.
10. Placement in rack: the robot places the opened tube into the next available position in the URIT analyzer rack, according to the cycle iteration number.
11. Rack handling: if the analyzer rack is full, the robot grips and loads the rack into the URIT urine analyzer</td>
</tr>
<tr>
<td>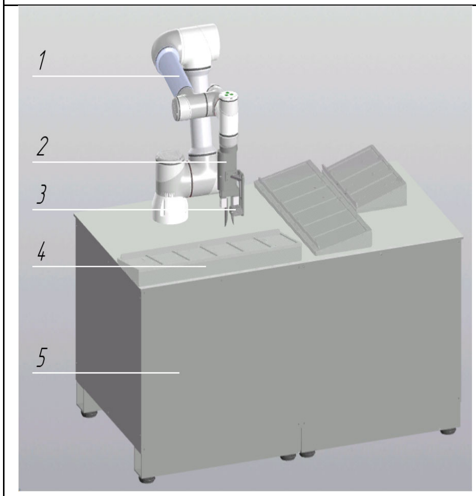
</td>
<td>Robotic Sorter</td>
<td>1. Connection verification: the stability of communication between the application and the active modules (robot, barcode scanner, and laboratory information system) is checked.
2. Robot initialization: the collaborative robot is initialized and moved to its starting position.
3. Tube selection and positioning: the robot selects the rack number and position within a standard 50-slot rack, positions itself above the tube, and grips the tube.
4. Tube presence verification: the system checks for the presence of the tube in the gripper. If the tube is absent, the gripper opens, and the robot moves to the next tube. In the absence of three consecutive tubes, the robot proceeds to the next rack.
5. Barcode scanning: if the barcode is missing or damaged, the tube is transferred to a dedicated rack for subsequent manual processing.
6. LIS query: based on the scanned barcode, a request with the sample identifier is sent to the laboratory information system (LIS).
7. LIS response waiting: the robot waits for a response from the LIS within a preset time limit. If the limit is exceeded, the tube is placed in a buffer rack.
8. Output rack selection: based on the received prioritization data for sample distribution across test panels, the output rack number and the tube’s internal position are selected.
9. Tube placement: the tube is moved to the designated position in the output rack.
10. Buffer monitoring: an automatic check is performed for LIS responses to tubes in the buffer rack. If a response is received, the tube is retrieved from the buffer, and the algorithm proceeds to step 8.
11. Cycle repetition: the process repeats for all tubes in all input racks</td>
</tr>
<tr>
<td>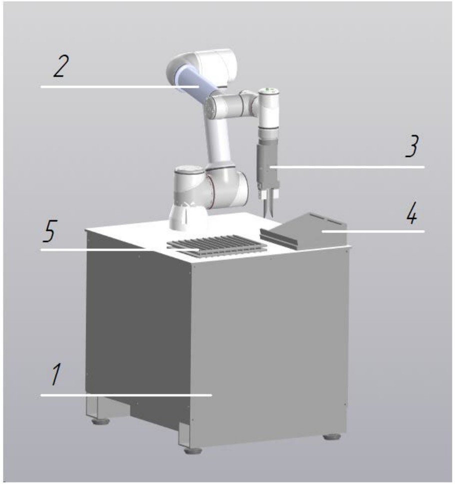
</td>
<td>Mindray BC Robotic Loader</td>
<td>1. Robot initialization: the collaborative robot is initialized and moved to its starting position.
2. Movement to the closed tube: the robot approaches a closed microtube.
3. Grip closure: the movable gripper pins close around the tube upon robot command.
4. Tube presence verification: the system checks for the presence of the tube in the gripper. If the tube is absent, the gripper opens, and the robot moves to the next tube. In the absence of two consecutive tubes, the robot proceeds to the next rack or ends the cycle.
5. Sample mixing: the robot mixes the tube contents by rotating and inverting it.
6. Placement in rack: the robot places the opened tube into the next available position in the Mindray analyzer rack, according to the cycle iteration number.
7. Rack handling: if the analyzer rack is full, the robot grips and loads the rack into the Mindray hematology analyzer</td>
</tr>
</table>
